# CFTR Modulators Dampen *Aspergillus*-Induced Reactive Oxygen Species Production by Cystic Fibrosis Phagocytes

**DOI:** 10.3389/fcimb.2020.00372

**Published:** 2020-07-24

**Authors:** Alexander J. Currie, Ellen T. Main, Heather M. Wilson, Darius Armstrong-James, Adilia Warris

**Affiliations:** ^1^Aberdeen Fungal Group, Institute of Medical Sciences, University of Aberdeen, Aberdeen, United Kingdom; ^2^Medical Research Council Centre for Medical Mycology, University of Exeter, Exeter, United Kingdom; ^3^Department of Infectious Diseases, Imperial College London, London, United Kingdom

**Keywords:** *Aspergillus fumigatus*, cystic fibrosis, phagocytes, inflammation, CFTR modulators, azithromycin, acebilustat

## Abstract

Excessive inflammation by phagocytes during *Aspergillus fumigatus* infection is thought to promote lung function decline in CF patients. CFTR modulators have been shown to reduce *A. fumigatus* colonization *in vivo*, however, their antifungal and anti-inflammatory mechanisms are unclear. Other treatments including azithromycin and acebilustat may dampen *Aspergillus*-induced inflammation due to their immunomodulatory properties. Therefore, we set out in this study to determine the effects of current CF therapies on ROS production and fungal killing, either direct or indirect by enhancing antifungal immune mechanisms in peripheral blood immune cells from CF patients upon *A. fumigatus* infection. Isolated peripheral blood mononuclear cells (PBMCs) and polymorphonuclear cells (PMNs) from CF patients and healthy volunteers were challenged with *A. fumigatus* following pre-treatment with CFTR modulators, azithromycin or acebilustat. Ivacaftor/lumacaftor treated CF and control subject PMNs resulted in a significant reduction (*p* < 0.05) in *Aspergillus*-induced ROS. For CF PBMC, *Aspergillus*-induced ROS was significantly reduced when pre-treated with ivacaftor alone (*p* < 0.01) or in combination with lumacaftor (*p* < 0.01), with a comparable significant reduction in control subject PBMC (*p* < 0.05). Azithromycin and acebilustat had no effect on ROS production by CF or control subject phagocytes. None of the treatments showed an indirect or direct antifungal activity. In summary, CFTR modulators have potential for additional immunomodulatory benefits to prevent or treat *Aspergillus*-induced inflammation in CF. The comparable effects of CFTR modulators observed in phagocytes from control subjects questions their exact mechanism of action.

## Introduction

Cystic fibrosis (CF) is a life-limiting autosomal recessive disorder characterized by chronic respiratory infections, progressive respiratory disease and respiratory failure (Elborn, 2016). Gene mutations in the cystic fibrosis transmembrane conductance regulator (CFTR) in epithelial cells affects mucus fluid dynamics and pathogen survival (Elborn, 2016; McElvaney et al., 2019). CFTR is also expressed in immune cells and mutations in this gene are associated with impaired antimicrobial activity and dysregulated inflammatory responses (Moss et al., 2000; Carrabino et al., 2006; Painter et al., 2006; Deriy et al., 2009; Del Porto et al., 2011; Mueller et al., 2011; Zhou et al., 2013; Johansson et al., 2014). Treatment of the bacterial infectious complications in CF patients has traditionally focused on clearance and eradication of the pathogen from the airways, thereby diminishing and preventing airway inflammation (Döring et al., 2012; Ciofu et al., 2013; Addy et al., 2020).

Airway inflammation in CF is a much-debated topic, as underlying mechanisms may be either intrinsic or extrinsic, or a combination of both (Nichols and Chmiel, 2015). Modulating the inflammatory response needs to consider a careful balance aimed at a minimum of inflammation without reducing the antimicrobial activity of the immune system. Management approaches to *Aspergillus* infections in CF have focused on Allergic Bronchopulmonary Aspergillosis (ABPA) by reducing the allergic inflammation induced (Agarwal et al., 2016). First-line treatment with corticosteroids is targeted against the induced inflammation, with a recommendation for antifungal therapy when first-line treatment fails. *Aspergillus fumigatus* is the major fungal pathogen isolated from sputum of far more CF patients than those diagnosed with ABPA (Warris et al., 2019). Studies indicate that infection with *A. fumigatus* in the CF airways can result in increased pulmonary exacerbations, bronchiectasis, and worse respiratory quality of life (Amin et al., 2010; Breuer et al., 2019; Hong et al., 2020). Nevertheless, there is a lack of data on how to manage those infections, e.g., eradication of infection or damping the inflammation, and what the associated risks and benefits of such approaches would be.

Our group previously demonstrated that peripheral blood phagocytes from CF patients show normal antifungal killing in response to *A. fumigatus*, but that this is associated with excessive reactive oxygen species (ROS) production (Brunel et al., 2018). Additionally, we highlighted that the heightened inflammation was correlated with poorer lung function in CF patients. As eradication of *A. fumigatus* from the airways of CF patients is a huge challenge due to the universal presence of *A. fumigatus* in the environment, finding ways to dampen *Aspergillus*-induced inflammation may be more feasible, as long as these strategies do not affect antifungal killing mechanisms.

In our current study, a key objective was to determine in detail the effects of three important CF therapies, azithromycin, acebilustat, and CFTR modulators, on the antifungal immune mechanisms. Azithromycin is a macrolide antibiotic with immunomodulatory and anti-inflammatory properties (Cigana et al., 2006; Legssyer et al., 2006) used as a long-term treatment for CF patients to improve lung function and reduce exacerbations in those with persistent *Pseudomonas aeruginosa* (Mogayzel et al., 2013; Principi et al., 2015). Acebilustat, currently in phase I/II trials in CF patients, is a leukotriene A4 hydrolase (LTA_4_H) inhibitor, inhibiting the production of the intracellular lipid mediator leukotriene B4 (LTB4) (Bhatt et al., 2017; Elborn et al., 2017a,b, 2018). LTB4 is a principal chemoattractant for recruiting neutrophils to inflamed sites across the airway epithelium and known to stimulate ROS production and to enhance the NF-κB pathway, thus driving inflammation (Woo et al., 2003). CFTR modulators are the first causative treatment option for CF and have been shown to reduce pulmonary exacerbations in CF patients homozygous for the F508-del mutation (Wainwright et al., 2015). Additionally, ivacaftor reduced colonization and prevalence of *A. fumigatus* in CF patients with a G551D genotype (Heltshe et al., 2015; Frost et al., 2019). Whilst the effects of CFTR modulators on epithelial cell function are reasonably well-understood (Kuk and Taylor-Cousar, 2015), the effects on immune cell function have not been investigated in detail. Assessment of the hypothetical effect of the CFTR modulators on immune cells resulting in a decrease of microbial induced inflammation, is of high value. We present here our results of the effect of these treatments on *Aspergillus*-induced ROS production and fungal killing, either direct or indirect by enhancing antifungal immune mechanisms in peripheral blood phagocytes.

## Materials and Methods

### Human Subjects

Blood samples were donated by adult CF patients attending the Aberdeen Royal Infirmary (Aberdeen, UK) and healthy volunteers recruited from the Institute of Medical Sciences (Aberdeen, UK). All participants provided written informed consent and donated a maximum of 50 mL (CF patients) or 100 mL (healthy volunteers) of blood. This study was approved by East of Scotland Research Ethics Service (18/ES/0154) and the College Ethics Review Board of the University of Aberdeen (CERB/2016/8/1300). All samples were collected according to approved guidelines and procedures. Clinical report forms were provided for each CF patient and included; demographics, genotype, body mass index (BMI), forced expiratory volume in 1 s (FEV_1_), *Aspergillus* serology, sputum culture results, co-morbidities, pulmonary exacerbation episodes over the previous 12 months and medications.

### *A. fumigatus* Strains

Thirteen *A. fumigatus* strains were used including 12 clinical strains and the well-characterized laboratory strain AF 293. Clinical isolates from CF patients (10,749, 11,361, 5,923, 7,762, 10,225, 15,115, 10,410, 11,856), patients with chronic infection (1,145, 9,475) and acute infection (11,146, 11,160) were kindly provided by Prof Paul E. Verweij (Centre for Expertise in Mycology, Radboud University Medical Centre, Nijmegen, NL).

### *A. fumigatus* Culture Conditions

*A. fumigatus* conidia were grown on glucose minimal media for 7 days at 35°C and harvested in phosphate buffer saline (PBS) supplemented with 0.05% Tween-80. Conidia were then filtered through a 40 μm sterile filter and resuspended to the required concentration in RPMI or PBS + Ca^2+^/Mg^2+^ (0.9 nM Ca^2+^ and 0.49 mM Mg^2+^).

### CFTR Modulators, Azithromycin, and Acebilustat

Ivacaftor and lumacaftor (AdooQ Bioscience, USA) stock solutions were prepared by solubilizing in 100% DMSO (Sigma Aldrich, UK) at 10 mg/mL. CFTR modulator stock solutions (1.6, 3.2, or 6.4 μl) were diluted in 1 mL sterile water. DMSO diluted in sterile water was used as a control in all experiments, at 0.32 or 0.64% when testing direct antifungal activity and 0.16% for ROS assays. Azithromycin dihydrate (Sigma Aldrich, UK) was solubilised in 100% DMSO at 20 mg/mL and stock solutions (1, 2, and 2.5 μl) were diluted in 1 mL of sterile water for ROS assays and RPMI for fungal killing. Acebilustat (MedChemExpress, USA) was solubilised in 100% DMSO at 0.1 mM and diluted to a working concentration of 10 μM in sterile water for ROS assays and RPMI for fungal killing.

Concentrations used in the various experiments were based the IC50 of acebilustat to inhibit LTB4 production, the maximum reported tissue concentrations of azithromycin, and previous *in vitro* studies performed with the CFTR modulators (reviewed by Csanády and Töröcsik, 2019).

### Phagocyte Isolation

Whole blood samples were collected in a Vacuette® containing EDTA (Greiner Bio-One) and allowed to cool to room temperature before mixing 1:1 with sterile PBS. Blood was then overlaid on equal parts of Histopaque (10,771 and 11,919; Sigma Aldrich) and separation of the cell fractions was achieved by density gradient centrifugation at 300 g for 30 min at 4°C. The peripheral blood mononuclear cell (PBMC) layer was removed and washed three times in PBS. The fraction containing polymorphonuclear cells (PMN) was treated twice with hypotonic lysis buffer (8.3 mg/ml NH_4_Cl and 1 mg/ml KHCO_3_ in sterile water) to lyse erythrocytes then washed three times with decreased spins to remove thrombocytes. PBMC and PMN pellets were resuspended in RPMI or PBS + Ca^2+^/Mg^2+^, counted and adjusted to the required concentration. Viability was tested by using trypan blue exclusion.

### Reactive Oxygen Species (ROS) Production

Production of oxygen radicals was evaluated using luminol-based chemiluminescence as previously described (Brunel et al., 2018). Briefly, PBMCs and PMNs were suspended in PBS +Ca^2+^/Mg^2+^ and plated at 5 × 10^5^ cells/well and left untreated (vehicle control) or pre-treated with ivacaftor (8 μg/ml), lumacaftor (8 μg/ml), ivacaftor+lumacaftor (8 μg/ml of each), acebilustat (0.031 μg/ml) or azithromycin (20 μg/ml) for 1 h at 37°C, 5% CO_2_. Cells were then infected with *A. fumigatus* resting conidia (1 × 10^7^/well) and 100 μM luminol was added to each well. Kinetic reads were taken every 180 s for 2 h using a luminescence plate reader (Biotek Gen5™).

### Fungal Killing

To assess direct fungal killing of each drug, *A. fumigatus* strains were plated at 5 × 10^4^ conidia per well in RPMI in a 96 U-well plate. Conidia were then incubated with ivacaftor/lumacaftor (16 or 32 μg/ml of each), azithromycin (10, 20, or 50 μg/mL), or acebilustat (0.031 μg/ml) for 18 h or left untreated (vehicle control). To test anti-hyphal activity; conidia were plated and left for 16 h (37°C, 5% CO_2_) to allow for gemination prior to incubation with ivacaftor (16 or 32 μg/ml), lumacaftor (16 or 32 μg/ml), or ivacaftor+lumacaftor (16 or 32 μg/ml of each) for 6 h.

To assess enhanced fungal killing by phagocytes, PMNs and PBMCs were plated at 1 × 10^5^ and 5 × 10^5^ cells per well respectively, and left untreated (vehicle control) or pre-treated with ivacaftor (8 μg/ml), lumacaftor (8 μg/ml), ivacaftor/lumacaftor (8 μg/ml of each), acebilustat (0.031 μg/ml) or azithromycin (20 μg/ml) for 1 h at 37°C, 5% CO_2_. Cells were then infected with *A. fumigatus* conidia (1 × 10^5^ conidia per well) and left for 18 h at 37°C, 5% CO_2_.

Following the indicated incubation times, plates were centrifuged at 2,500 g for 10 min. For phagocyte experiments, media was removed, and cells lysed with 100 μl of saponin (0.005% in MilliQ water; Sigma Aldrich) for 20 min. For the cell-free experiments, fresh media was added. Twice concentrated XTT-menadione solution (XTT salt 200 μg/ml, Invitrogen; menadione crystalline 172 μg/ml, Sigma Aldrich) was added to each well (100 μl diluted 1:2 in media or saponin) and plates left for 2–3 h in the dark at 37°C, 5% CO_2_ to allow for reduction of XTT to formazan. Plates were spun at 2,500 g for 10 min and supernatant transferred to a flat-bottomed 96-well plate prior to measuring absorbance at 450 nm using a VersaMax microplate reader.

### Statistical Analysis

All data are presented as mean ±SEM. Significance between control subjects and CF patients was analyzed using Mann Whitney *U*-tests. For multiple comparisons between drug treatments a Kruskal–Wallis test was used with Dunn's post-test. Data analysis was carried out using GraphPad Prism V5.04.

## Results

### Clinical Characteristics of Participants

Ten patients (60% male) participated in this study with a median age of 26 years (range 16–46 years). Six were homozygous for the F508-del mutation. Median FEV_1_ was 43.5% predicted (range 15–104.2%) and BMI was 21 (range 17–40). None of the patients received CFTR modulators or antifungals. Four patients had signs of fungal sensitization (*Aspergillus* IgE > 1 kU/L) (Supplementary Table 1). Control subjects were between the ages of 20–55 years.

### CFTR Modulators and Azithromycin Have no Direct Antifungal Effect

**Figure 1:**
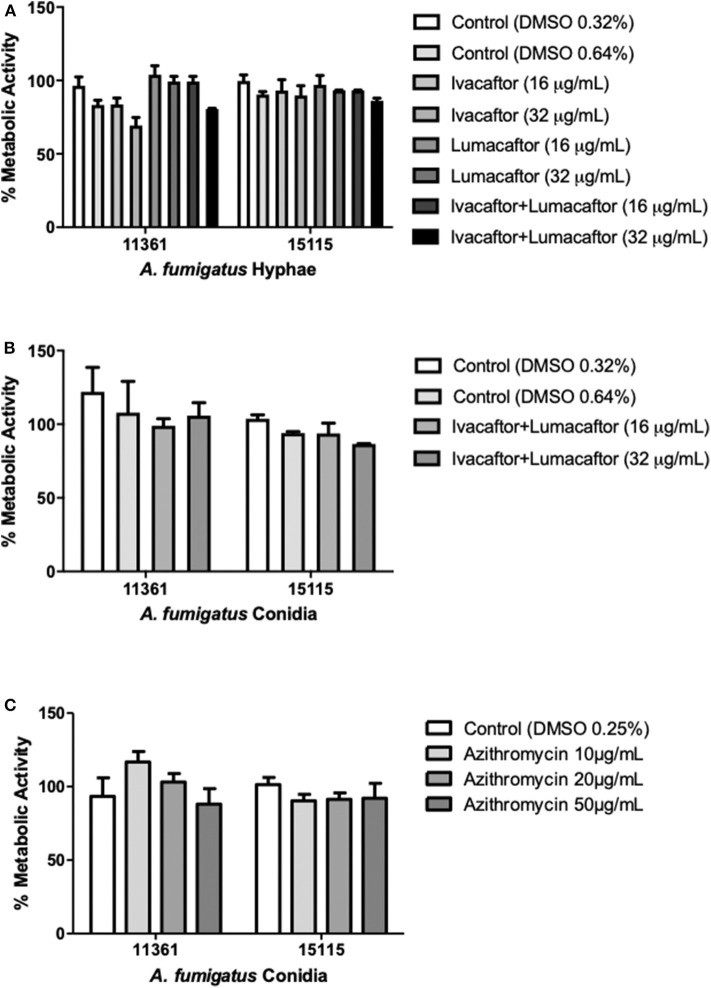
CFTR modulators and azithromycin have no direct antifungal effect. **(A)** Hyphae from 2 clinical *A. fumigatus* isolates (11,361, 15,115) were untreated (DMSO) or treated with ivacaftor (16 or 32 μg/ml), lumacaftor (16 or 32 μg/ml) or ivacaftor/lumacaftor (16 or 32 μg/ml of each) for 6 h prior to measuring metabolic activity. **(B)** Two clinical *A. fumigatus* isolates (11,361, 15,115) were grown in the presence of DMSO or ivacaftor/lumacaftor (16 or 32 μg/ml of each) or **(C)** in the presence of various concentrations of azithromycin for 18 h at 37°C, 5% CO_2_. Following the specified incubations metabolic activity was assessed with XTT-menadione. Data are representative of 2 **(A,B)** and 3 **(C)** independent experiments and are presented the mean ±SEM of % metabolic activity compared to *A. fumigatus* grown in RPMI only.

Ivacaftor (16 or 32 μg/ml), lumacaftor (16 or 32 μg/ml), or ivacaftor/lumacaftor (16 or 32 μg/ml of both) did not have a significant effect on the hyphal metabolic activity for both *A. fumigatus* isolates when compared to the vehicle controls (Figure 1A). No direct effect was observed on the metabolic activity of an additional 10 clinical isolates and one lab strain (AF293) with all concentrations and combination tested (Supplementary Figure 1). When incubating *A. fumigatus* conidia with ivacaftor/lumacaftor (16 or 32 μg/ml of both), no effect on the metabolic activity was observed when compared to the vehicle controls (Figure 1B). Again, no effect was observed for an additional 10 clinical isolates and one lab strain (AF293) (Supplementary Figure 2). No effect on fungal metabolic activity was observed for all azithromycin concentrations tested (Figure 1C, Supplementary Figure 3).

### *Aspergillus* Activated Phagocytes From CF Patients Show Exaggerated ROS Production

**Figure 2:**
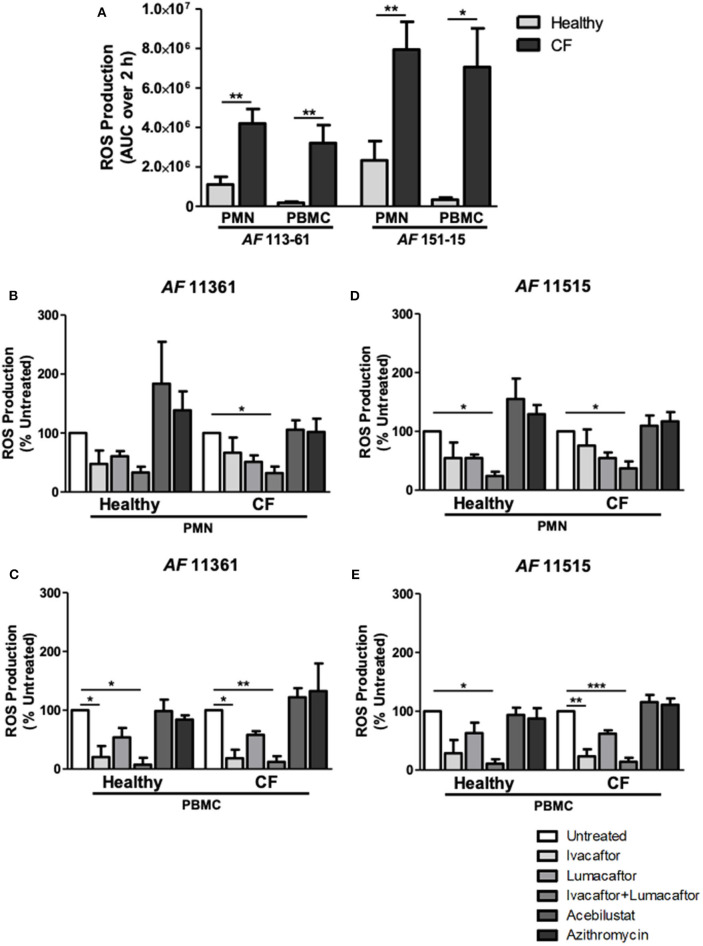
CFTR modulators, but not acebilustat or azithromycin, reduce *Aspergillus-*induced ROS production by both healthy and CF phagocytes. Isolated PMN or PBMC were either untreated (0.16% DMSO) or treated with ivacaftor (8 μg/ml), lumacaftor (8 μg/ml), ivacaftor/lumacaftor (8 μg/ml of both), acebilustat (0.031 μg/ml) or azithromycin (20 μg/ml) for 1 h at 37°C, 5% CO_2_ prior to *A. fumigatus* infection (MOI 20). ROS was measured by luminol chemiluminescence. **(A)** ROS production by control and CF PMN and PBMC in response to *A. fumigatus* CF patient isolates 11,361 and 15,115. **(B–D)** ROS production by control and CF PMN in response to two *A. fumigatus* isolates originating from CF patients following pre-treatment with ivacaftor, lumacaftor, acebilustat or azithromycin and **(C–E)** and by control and CF PBMC. Data presented relative to untreated samples which was set as 100%. Data are presented as mean ±SEM of total ROS produced over 2 h (AUC) for 4–5 control subjects and 5–9 CF patients. Statistical analysis was carried out using **(A)** Mann-Whitney test and **(B–E)** Kruskal-Wallis test. Abbreviations, AUC; area under the curve, PMN; polymorphonuclear cells, PBMC; peripheral blood mononuclear cells, ROS; reactive oxygen species, SEM; standard error of the mean. *p ≤ 0.05; **p ≤ 0.001; ***p ≤ 0.001.

Phagocytes from CF and control subjects were co-incubated with *A. fumigatus* conidia and analyzed for ROS production. ROS production after incubation with two different clinical isolates of *A. fumigatus* conidia was significantly increased by CF PMN and PBMC when compared to healthy controls (Figure 2A) confirming our previous observations (Brunel et al., 2018). CF PMN produced ROS at levels up to 4-fold greater than PMNs from control subjects in response to both *A. fumigatus* isolates (*p* ≤ 0.01). *A. fumigatus*-induced ROS production by CF PBMC was 18- to 20-fold higher (*p* ≤ 0.01) when compared to cells from control subjects (Figure 2A).

### CFTR Modulators Reduce *Aspergillus*-Induced ROS by Phagocytes

The viability of the phagocytes with or without the CFTR modulators (alone or in combination at 8 μg/mL) remained around 95% after 6 h incubation. To assess the effect of each treatment on *Aspergillus*-induced ROS production by healthy and CF phagocytes, we normalized each treatment response as a percentage of the untreated control. A decrease in ROS production by CF PMN was observed following pre-treatment with ivacaftor (11,361; −33.4 ± 25.9%, 15,115; −24.1 ± 27.5%) and lumacaftor (11,361; −48.9 ± 11.01%, 15,115; −45.3 ± 9.5%) when compared to untreated controls, although the changes did not reach statistical significance. Ivacaftor/lumacaftor pre-treatment resulted in a significant reduction of *Aspergillus*-induced ROS production by CF PMNs for both strains (11,361; −67.8 ± 10.8%, 15,115; −62.8 ± 11.6%, *p* ≤ 0.05) (Figures 2B,D).

The same trend was observed in PMN from control subjects; ivacaftor (11,361; −52.5 ± 23.0%, 15,115; −45.3 ± 26.3%) and lumacaftor (11,361; −39.33 ± 8.7%, 15,115; −45.4 ± 6.0%) reduced ROS levels, although not statistically different from untreated PMN. When PMN from control subjects were pre-treated with ivacaftor/lumacaftor, this significantly reduced ROS production in response to both strains (11,361; −66.8 ± 9.7%, 15,115; −75.8 ± 7.1%, *p* ≤ 0.05) (Figures 2B,D).

For CF PBMC, *Aspergillus*-induced ROS was significantly reduced when pre-treated with ivacaftor (11,361; −81.7 ± 14.6%, *p* ≤ 0.05; 15,115; −76.60 ± 11.89%, *p* ≤ 0.01), but not when treated with lumacaftor (11,361; −41.9 ± 6.3%; 15,115; −38.2 ± 5.8%). Ivacaftor/lumacaftor pre-treatment significantly attenuated generation of ROS by CF PBMCs (11,361; −87.96 ± 9.7%, *p* ≤ 0.01; 15,115; −85.9 ± 6.7%, *p* ≤ 0.001) (Figures 2C,E).

As observed with CF PBMC, pre-treatment of control PBMC with ivacaftor showed a clear decrease in ROS production (11,361; −79.7 ± 14.6%, *p* ≤ 0.05; 15,115 −71.32 ± 22.4%, *p* = n.s.). Ivacaftor/lumacaftor significantly reduced ROS production by control subject PBMC (11,361; −92.5 ± 11.7%, 15,115; −89.14 ± 7.57%, *p* ≤ 0.05 for both strains) (Figures 2C,E).

**Figure 3:**
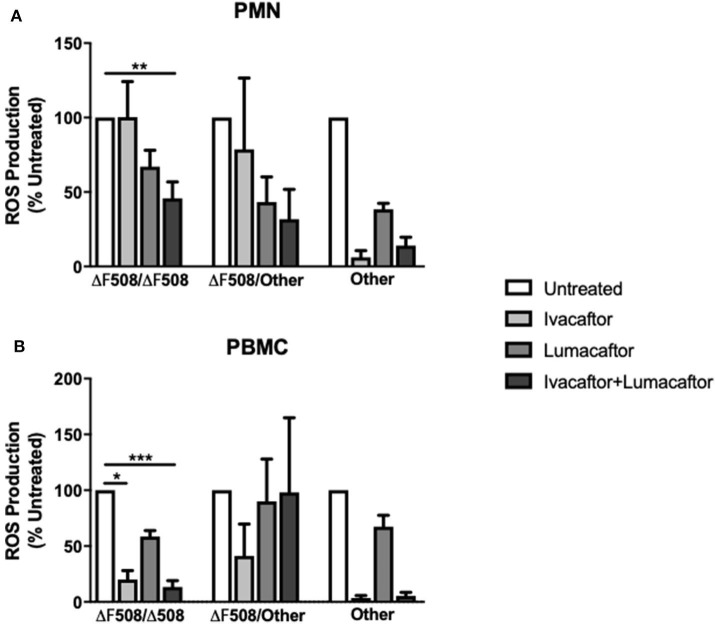
CFTR modulators differentially influence ROS production based on CFTR genotypes and type of immune cells. Isolated PMN or PBMC from CF patients with different underlying CFTR gene mutations were either untreated (0.16% DMSO) or treated with ivacaftor (8 μg/ml), lumacaftor (8 μg/ml), ivacaftor/lumacaftor (8 μg/ml of both) for 1 h at 37°C, 5% CO_2_ prior to *A. fumigatus* infection (MOI 20). ROS was measured by luminol chemiluminescence. **(A)** ROS production by CF PMN in response to two clinical *A. fumigatus* isolates from CF patients (11,361 and 15,115) following pre-treatment with ivacaftor, lumacaftor, and **(B)** by CF PBMC. Data presented relative to untreated samples which was set as 100%. Data are presented as mean ±SEM of total ROS produced over 2 h (AUC) for 5–9 CF patients. AUC, area under the curve; PMN, polymorphonuclear cells; PBMC, peripheral blood mononuclear cells; ROS, reactive oxygen species; SEM, standard error of the mean. *p ≤ 0.05; **p ≤ 0.001; ***p ≤ 0.001.

Next, we assessed the differential effects of those treatments based on underlying CFTR genotypes. PMN and PBMC from CF patients homozygous for the F508-del mutation (*n* = 6) treated with ivacaftor/lumacaftor showed a significant reduction in ROS (–54.2 ± 11.0%; *p* ≤ 0.01) and (−86.6 ± 5.7%; *p* ≤ 0.001, respectively) (Figure 3). Ivacaftor alone significantly reduced ROS production by PBMC homozygous for the F508-del mutation (−80.0 ± 8.1%; *p* ≤ 0.05), but had no effect on PMN (F508-del/F508-del). Lumacaftor pretreatment of PMN and PBMC resulted in a 33.0% (±11.1%) and 41.35% (±5.3%) reduction, respectively, in the F508-del homozygous group which was not significantly different to untreated cells (Figure 3).

Treatment with ivacaftor alone or in combination with lumacaftor showed a significant decrease in *Aspergillus*-induced ROS production by PMN (−93.8 ± 4.5 and −85.9 ± 5.6%, respectively) and PBMC (−96.4 ± 2.2 and −94.7 ± 3.2%, respectively) from CF patients with other CFTR mutations (non F508-del, non G551D) (Figure 3).

Pre-treatment with azithromycin (20 μg/ml) or acebilustat (64 nM) had no effect on *Aspergillus*-induced ROS production by PMN or PBMC from either CF patients or control subjects when compared to untreated cells (Figures 2B–E).

### CFTR Modulators, Acebilustat, and Azithromycin Do Not Impair Fungal Killing by Healthy and CF Phagocytes

**Figure 4:**
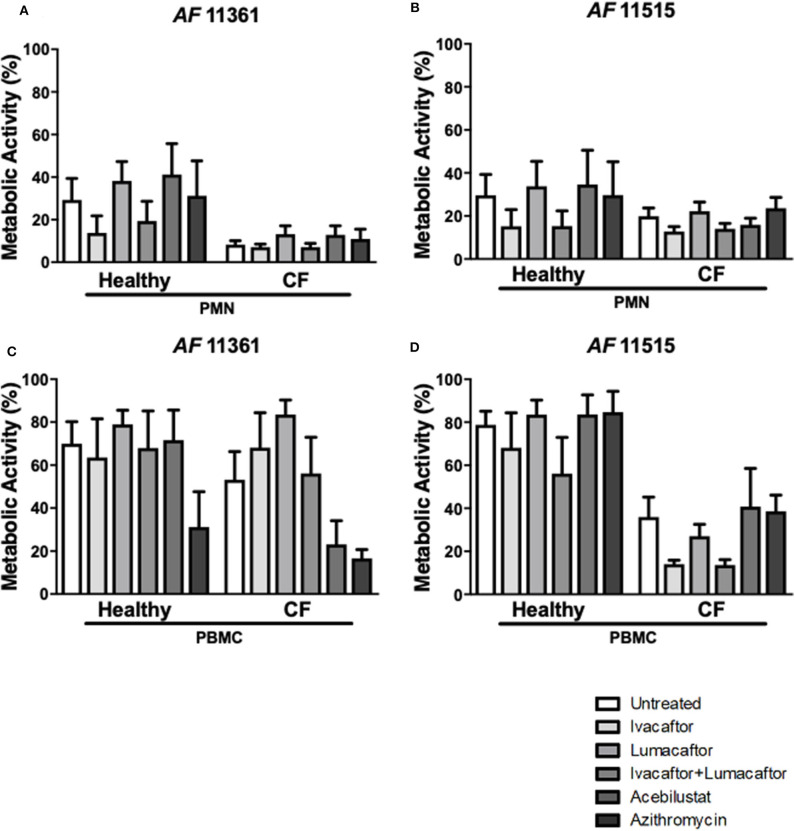
CFTR modulators, acebilustat and azithromycin do not impair fungal killing by healthy and CF phagocytes. Isolated PMN or PBMC were either untreated (0.16% DMSO) or treated with ivacaftor (8 μg/ml), lumacaftor (8 μg/ml), ivacaftor/lumcaftor (8 μg/ml of both), acebilustat (0.031 μg/ml) or azithromycin (20 μg/ml) for 1 h at 37°C, 5% CO_2_ prior to *A. fumigatus* infection (PMNs MOI: 1; PBMCs MOI: 0.2). Following 18 h incubation metabolic activity was determined using XTT-menadione. Assessment of fungal metabolic activity following co-incubation with pre-treated control and CF PMN **(A,C)** and PBMC **(B,D)** compared to untreated cells. Data are presented as mean ±SEM for 4–5 healthy controls and 3–6 CF patients. Statistical analysis was carried out using Kruskal–Wallis test. PMN, polymorphonuclear cells; PBMC, peripheral blood mononuclear cells; SEM, standard error of the mean.

Control subject PMN reduced metabolic activity by ~70% for both *A. fumigatus* isolates (Figures 4A,C). In comparison, CF PMN reduced the metabolic activity of the two isolates between 80 and 92% (Figures 4A,C). None of the treatments had any effect on killing of the two *A. fumigatus* isolates by both CF and control subject PMN when compared to untreated controls (Figures 4A,C). CF PBMC showed an increased killing of both isolates but changes did not reach statistical significance when compared to control subject PBMC (Figures 4B,D). No significant differences in antifungal killing were observed associated with a specific treatment given compared to untreated CF and control subject PBMC (Figure 4B).

## Discussion

We show that the CFTR modulators, ivacaftor, lumacaftor and its combination, are able to downregulate ROS production by human CF phagocytes without compromising their fungal killing ability. Importantly, this effect was not specific to CF cells, indicating potential off-target mechanistic effects of CFTR modulators. To our knowledge we are the first to demonstrate that CFTR modulators have immunomodulatory effects on both CF and control subjects' phagocytes when challenged with *A. fumigatus*. Azithromycin and acebilustat did not affect ROS production or fungal killing by CF or control subjects' phagocytes. Furthermore, CFTR modulators and azithromycin do not directly affect fungal viability.

Our study shows that CFTR modulators reduce ROS responses by human CF phagocytes infected with *A. fumigatus*, and that this reduction was statistically significant in PMN and PBMC from patients homozygous for the F508-del mutation treated with ivacaftor/lumacaftor. In addition, pretreatment of PBMC homozygous for the F508-del mutation with ivacaftor alone significantly reduced the *Aspergillus*-induced ROS production. As the number of CF patients heterozygous for the F508-del mutation and those with other mutations (non F508-del, non G551D) were low (both *n* = 2), a proper comparison between the three groups was not possible. Nevertheless, a comparable trend was observed for the phagocytes from CF patients heterozygous for the F508-del mutation. Remarkable is the observation that in the two CF patients with non-F508-del and non-G551D mutations, treatment with ivacaftor alone and in combination with lumacaftor almost completely abolished the *Aspergillus*-induced ROS production in PMN and PBMC. Most of the observed differences can be related to the specific mode of action of ivacaftor and lumacaftor. Lumacaftor acts directly to improve the defective cellular processing and trafficking of the F508-del mutant CFTR channel, with ivacaftor potentiating the gating properties of the mutant CFTR channel caused by a variety of gene mutations (Kuk and Taylor-Cousar, 2015).

The differential effect of ivacaftor pretreatment leading to a significant reduction in *Aspergillus*-induced ROS production by CF PBMC is in sharp contrast with the effect observed on CF PMN. A higher sensitivity of CF PBMC for potentiating the channel function might explain this observation, but needs further research. Improvements of ion fluxes underpinning the clinical efficacy of the CFTR modulators will likely influence the aberrant immune responses and observed hyperinflammation (Hartl et al., 2012; Pohl et al., 2014).

Only limited data is available showing that ivacaftor/lumacaftor can directly modulate CF-related inflammation. Studies performed with *P. aeruginosa* stimulated CF bronchial epithelial cells (homozygous for F508-del) showed that this combination reduces the transcription of CXCL8 and the phosphorylation of p38 MAPK (Ruffin et al., 2018). Human CF monocytes (homozygous for F508-del) stimulated with LPS/ATP before and after patients received treatment with ivacaftor/lumacaftor showed decreased levels of IL-18, TNF and caspase-1 (Jarosz-Griffiths et al., 2020). Excessive ROS production is linked to defective autophagy (Luciani et al., 2011). In CF epithelial cells, stockpiling of large amounts of mutant CFTR leads to increases in aggresomes and ROS production, and autophagy inhibition (Luciani et al., 2010). A comparable phenomenon has been observed in CF macrophages (Abdulrahman et al., 2013). CFTR modulators have been shown to have the ability to target autophagy in CF airway epithelial cells to decrease inflammation in the lung (Luciani et al., 2012) and might underpin the reduced ROS production as shown in our results.

Remarkably, ivacaftor and lumacaftor also decreased *Aspergillus*-stimulated ROS production by phagocytes from control subjects. Potentiation of the CFTR channel above normal physiological function or yet unknown off-target effects might explain this observation. It is important to acknowledge that the exact mechanisms of action have not been elucidated for both ivacaftor and lumacaftor.

Azithromycin did not affect the ROS production by CF or control subjects' phagocytes in response to *A. fumigatus*. Azithromycin has both immunomodulatory and anti-inflammatory properties (Cigana et al., 2006; Legssyer et al., 2006) and accumulates in neutrophils (Bosnar et al., 2005), but the effect on ROS production has hardly been studied. Bystrzycka et al. (2017) demonstrated a concentration-dependent effect of azithromycin (0.5–50 μg/ml) in decreasing the amount of ROS produced by PMA stimulated healthy human neutrophils. Earlier studies suggest that the effect of azithromycin depends on the stimulus used (Culić et al., 2002; Parnham et al., 2005). Due to the systemic glutathione deficiency in CF patients, azithromycin may be of value to improve the antioxidant activities by CF cells, thereby diminishing the toxic effects of ROS on lung tissue (Roum et al., 1993).

Acebilustat has not been investigated with respect to its influence on ROS production in immune cells. Based on the fact that LTB4 induces ROS production by dHL-60 neutrophils, inhibition of LTA4H by acebilustat would be predicted to diminish ROS production (Woo et al., 2003). However, acebilustat had no effect on ROS production by either CF or control subjects' phagocytes in our study. Its anti-inflammatory properties in the inflamed lung are most likely attributed to its inhibitory effects on neutrophil migration into the airways and the lungs (Woo et al., 2003).

Pretreatment with CFTR modulators was not associated with an enhanced fungal killing by CF and control subjects' phagocytes. This is in contrast with reports that CFTR modulators augment bacterial killing. Ivacaftor has been shown to augment killing of *P. aeruginosa* by CF macrophages (G551D/F508-del) to the same degree as healthy cells (Pohl et al., 2014). Similarly, lumacaftor alone increased killing of *P. aeruginosa* by CF macrophages (homozygous for Phe508del), but no effect was seen on control subjects' monocytes (Barnaby et al., 2018).

Azithromycin pre-treatment of healthy and CF phagocytes did not influence fungal killing. A different observation is reported for bacterial killing, as azithromycin-loaded neutrophils showed more effective killing of *Aggregatibacter* sp. by increased phagocytosis (Lai et al., 2015). Despite reports demonstrating that macrolide antibiotics have *in vitro* antifungal activity against *Aspergillus* species and other fungi (Kim et al., 2003; Hosoe et al., 2006), we did not observe any direct antifungal effect of azithromycin. Previous studies show long term azithromycin treatment can result in increased risk of colonization with *A. fumigatus* in CF patients possibly associated with its inhibitory effect on immune responses (Legssyer et al., 2006; Jubin et al., 2010). Additionally, there is an association between *A. fumigatus* colonization and non-tuberculous mycobacteria (NTM) in CF patients (Coolen et al., 2015). Although long term azithromycin reduces the risk of NTM, it does not suggest additional benefits to prevent *Aspergillus* infections.

Ivacaftor has been shown to have direct antibacterial effects against *Staphylococcus aureus* (MIC 8 μg/ml) and *Streptococcus* spp. (MIC 32 μg/ml), but was not active against *P. aeruginosa* (Reznikov et al., 2014). Similarly, Payne et al. (2017) showed 32 mg/L of ivacaftor resulted in a several log-fold decrease in CFUs with *Streptococcus* spp. and bacteriostatic effects against *S. aureus*, but was ineffective against *P. aeruginosa*. Clinical studies have shown ivacaftor reduces colonization of *A. fumigatus* in CF patients (with at least one copy of G551D mutation) and *P. aeruginosa*, but not *S. aureus* (Heltshe et al., 2015). Using data from the UK CF Registry comparing ivacaftor users and their contemporaneous comparators, reduced prevalence of *Aspergillus* spp., as well as *P. aeruginosa* and *S. aureus*, but not *Burkholderia cepacia* were found (Frost et al., 2019). We show that ivacaftor and/or lumacaftor have no antifungal activity, suggesting that reduced colonization observed clinically is not due to a direct effect. Synergy in bacterial killing by combining CFTR modulators and specific antimicrobials indicates possible additional benefit to treat CF lung infections. Schneider et al. (2016) found that using ivacaftor or ivacaftor/lumacaftor in combination with polymyxin B increases killing of *P. aeruginosa*. Ivacaftor in combination with vancomycin or ciprofloxacin increased the potency of these antibiotics against *S. aureus* and *P. aeruginosa*, respectively (Reznikov et al., 2014). Comparable studies with antifungal drugs are lacking. However, for the most commonly used antifungals, the mold-active azoles, an extra challenge is faced when co-prescribing these two drugs due to drug-drug interactions (Jordan et al., 2016).

In summary, CFTR modulators may have additional immunomodulatory benefits to prevent or treat *Aspergillus*-induced inflammation in CF. The comparable effects of CFTR modulators observed in phagocytes from control subjects questions their exact mechanism of action.

## Data Availability Statement

The raw data supporting the conclusions of this article will be made available by the authors, without undue reservation.

## Ethics Statement

The studies involving human participants were reviewed and approved by East of Scotland Research Ethics Service (18/ES/0154) and the College Ethics Review Board of the University of Aberdeen (CERB/2016/8/1300). The patients/participants provided their written informed consent to participate in this study.

## Author Contributions

AW and DA-J conceived and designed the study. AC and EM performed the experiments and analyzed the data. HW provided expertise and analysis support. AC, AW, and HW wrote the manuscript. All authors contributed in the revision of the manuscript and approve of submission.

## Conflict of Interest

The authors declare that the research was conducted in the absence of any commercial or financial relationships that could be construed as a potential conflict of interest.
