# Supplementary material for: CFTR Modulators Dampen Aspergillus-Induced Reactive Oxygen Species Production by Cystic Fibrosis Phagocytes
Source: Front Cell Infect Microbiol. 2020 Jul 24;10:372. doi: 10.3389/fcimb.2020.00372 (PMC7393064; doi:10.3389/fcimb.2020.00372)
Supplement: Supplementary file 1 [file Data_Sheet_1.docx]

CFTR modulators dampen *Aspergillus*-induced reactive oxygen species production by cystic fibrosis phagocytes.

Supplementary Material

**Table 1.** Patient characteristics (n=10).

| Variables |  |  |  |
| --- | --- | --- | --- |
| Age, median (range), years | | | 26 (16-46) |
| Sex, male | | | 6 (60%) |
| BMI (range) | | | 21 (17-40) |
| FEV_1_, median (range), % predicted | | | 43.5 (15-104.2) |
| F508del homozygous | | | 6 (60%) |
| Medications   - Macrolides - Antifungals - Inhaled Anti-*Pa* Therapy - Inhaled Corticosteroids - Oral Steroids - CFTR Modulators | | | 6 (60%)  0  6 (60%)  5 (50%)  3 (30%)  0 |
| Pulmonary exacerbations,  previous 12 months, median (range) | | | 3 (1-14) |
| Days hospitalised,  previous 12 months,  median (range) | | | 26 (3-77) |
| CF-related Diabetes | | | 5 (50%) |
| Sputum microbiology   - Chonic *Pa* colonised - *Aspergillus* in sputum,   previous 12 months | | | 4 (40%)  0 |
| Aspergillus IgE > 1 kU/L | | | 4 (40%) |
| Aspergillus IgG mg/L, median(range) | | | 47.7 (21-82) |

Abbreviations; BMI – body mass index, FEV_1_ - forced expiratory volume in 1 s, *Pa* – *Pseudomonas aeruginosa*.

**Supplementary figure 1. CFTR modulators have no direct antifungal effect on *A. fumigatus* hyphae.** *A. fumigatus* isolates from CF patients, acute and chronic infections and a lab strain, were allowed to germinate for 18 h at 37°C, 5% CO_2_. Hyphae were then treated with DMSO control or ivacaftor (16 or 32 µg/ml), lumacaftor (16 or 32 µg/ml) or ivacaftor+lumacaftor (16 or 32 µg/ml of each) for 6 h prior to measuring metabolic activity with XTT-menadione. Data are representative of 2 independent experiments and are presented as the mean ± SEM of % metabolic activity compared to *A. fumigatus* grown in RPMI only.

**Supplementary figure 2. CFTR modulators have no direct antifungal effect on *A. fumigatus* conidia.** *A. fumigatus* conidia from CF patient isolates, acute and chronic infections and a lab strain were grown in the presence of DMSO control or ivacaftor (16 or 32 µg/ml), lumacaftor (16 or 32 µg/ml) or ivacaftor+lumacaftor (16 or 32 µg/ml of each) for 18 h at 37°C, 5% CO_2_, prior to measuring metabolic activity with XTT-menadione. Data are representative of 2 independent experiments and are presented as the mean ± SEM of % metabolic activity compared to *A. fumigatus* grown in RPMI only.

**Supplementary figure 3. Azithromycin has no direct antifungal effect on *A. fumigatus* conidia.** *A. fumigatus* conidia from CF patient isolates, acute and chronic infections and a lab strain were grown in the presence of DMSO control or 10, 20 or 50µg/ml of azithromycin for 18 h at 37° C, 5% CO_2_, prior to measuring metabolic activity with XTT-menadione. Data are representative of 3 independent experiments and are presented the mean ± SEM of % metabolic activity compared to *A. fumigatus* grown in RPMI only.
